# Pilot study comparing the two hemostatic agents in patients undergoing partial nephrectomy

**DOI:** 10.1186/1756-0500-6-399

**Published:** 2013-10-03

**Authors:** Diego Aguilar Palacios, Michael McDonald, Makito Miyake, Charles J Rosser

**Affiliations:** 1 Section of Urologic Oncology, MD Anderson Cancer Center Orlando, Orlando, FL 32806, USA; 2 Department of Urology, Celebration Hospital – Florida Hospital, Celebration, FL 34747, USA; 3 Cancer Research Institute, Orlando Health, 6900 Lake Nona Blvd, Orlando, FL 32527, USA

**Keywords:** Haemostatic agent, Nephrectomy, Nephron-sparing, Partial, Renal mass

## Abstract

**Background:**

Recently studies have demonstrated improved outcomes in patients undergoing nephron-sparing surgery (NSS) for low stage renal tumors, thus NSS is widely accepted as the treatment option for these patients. With NSS, there is a risk of renal hemorrhage and thus haemostatic agents may be routinely applied to the cut surface of the kidney. Herein we compare two commercially available haemostatic agents applied intra-operatively to the cut surface of the kidney. Post-operative outcomes (oncologic and non-oncologic) are reported.

**Methods:**

The medical records of 23 patients with suspicious renal mass documented on axial imaging and who underwent open NSS via a mini-subcostal incision were extensively reviewed. One of two haemostatic agents (Floseal^®^, n = 11; Arista^®^, n = 12) was intra-operatively applied to the cut surface of the kidney. Chi-square and T- student test was used to compare outcomes between the cohort of 11 patients who had Floseal^®^ and the 12 patients who had Arista^®^.

**Results:**

Median pre-operative size of renal mass was 4.3 cm (range 1.5-7.0 cm). Final pathology revealed 3 oncocytomas and 20 renal cell carcinoma (17 clear cell, 1 chromophobe and 2 papillary), pT1a = 14 and pT1b = 6. Mean intra-operative blood loss and hospital stay between the Floseal^®^*vs*. Arista^®^ cohorts did not significantly differ (227 mL *vs*. 250 mL, *p* = 0.68 and 4.4 days *vs*. 4.5 days, *p* = 0.76, respectively). Intra-operative and post-operative complications were not different between the two cohorts. No recurrences have been documented with a mean follow-up of 18 months.

**Conclusion:**

Along with meticulous surgical technique, the use of either haemostatic agent (Floseal^®^ or Arista^®^) was not associated with high rate of intra-operative or post-operative haemorrhage. Thus either haemostatic agent may be successfully used during NSS.

## Background

In 2012, it is estimated that 64,770 Americans will be diagnosed with cancer of the kidney resulting in approximately 13,570 deaths [1]. Interestingly, kidney cancer is one of only a few cancers with an increasing incidence over the past two decades [2]. Renal cell carcinoma (RCC) accounts for over 85% of all kidney tumors, which makes up 2-3% of all adult malignancies [3]. Approximately, 70% of RCCs are incidentally discovered on axial imaging of the abdomen with >50% of RCCs being low-stage (T1-T2 N0M0) [4,5]. Despite advancements in drug discovery for advanced RCC, mortality rates have not changed over the past two decades [6], however for patients with low-stage disease, surgical extirpation offers excellent 5-year survival rates of 95% [5].

Traditionally, surgical expiration of these low-stage tumors was in the form of radical nephrectomy (*i*.*e*., removal of kidney, Gerota’s fascia and ipsilateral adrenal gland) [7]. Then contemporary studies demonstrate that in select tumors, adrenal sparing surgery [8] and nephron sparing surgery (NSS) [9] could be effectively performed without compromising oncologic outcomes. Recently studies have demonstrated improved non-oncological outcomes in patients undergoing NSS for low-stage tumors [10]. Thus, we currently find ourselves in the midst of a new era in the management of low-stage kidney cancer, where NSS is center stage and widely accepted as the treatment option for these patients.

It is not unusual to use haemostatic agents during procedures with a propensity for bleeding. NSS is one of these procedures. Numerous haemostatic agents are available for use. One such agent that has been widely used over the past decade is Floseal^®^. Floseal^®^ consists of patented bovine-derived gelatin granules coated in human-derived thrombin that work in combination to form a stable clot at the bleeding site [11]. Floseal^®^, FDA approved in 1999, expands approximately 20% within about 10 minutes, giving predictable control during and after surgery [12]. Another haemostatic agent, Arista^®^, is a synthetic haemostatic compound that includes an absorbable haemostatic powder consisting of Microporous Polysaccharide Hemospheres (MPH^®^). Arista works through accelerating the intrinsic clotting cascade by extracting fluid from blood, concentrating serum proteins and platelets, which is the scaffolding for the formation of a fibrin clot [13].

Earlier studies reported relatively high complication rates associated with NSS [14], which have greatly reduced over the past decade due to surgical refinement [15,16]. One such complication was haemorrhage related to the surgery (intrao-perative or post-operative). Thus the successful incorporation of a haemostatic agent, which may be routinely applied to the cut surface of the kidney, may prove to be advantageous. Herein we compare two commercially available haemostatic agents applied intra-operatively. Intra-operative and post-operative outcomes (oncologic and non-oncologic) are reported.

## Method

After MD Anderson Cancer Center Orlando institutional review board approval with consent waiver, medical records of patients who had undergone NSS for a suspicious renal mass from December 2009 to September 2012 were extensively reviewed. Preferentially, non-hilar, renal lesions < 7 cm were considered for NSS. Using these selection criteria, our study cohort was comprised of 23 patients who underwent NSS. Clinical and hospital records were reviewed for demographics, smoking history, body mass index (BMI), comorbidities, pre-operative and post-operative glomerular filtration rate (GFR), intra-operative features, disease characteristics and outcomes (oncologic and non-oncologic).

All patients had renal masses visualized and characterized by axial imaging (CT scan = 20, MRI scan = 2 and CT scan/MRI scan = 1). Of the 23 patients with renal masses, 3 renal masses were noted to be cystic masses (Bosniak III = 2, size 3 cm and 5 cm and Bosniak IV = 1, mean size 5.5 cm), while the remainder was solid. Approximately four weeks prior to NSS, patient had serum creatinine drawn and GFR was calculated. In three of the 23 patients, a percutaneous core biopsy of the renal mass was performed demonstrating RCC (Clear cell carcinoma = 2 and papillary carcinoma = 1). All patients underwent open mini-incision NSS.

Open NSS was performed as previously described [17] with modification by one surgeon (CJR). Briefly, the renal artery was clamped and renal parenchyma was transected and the tumor excised with grossly negative surgical margins. The surgical specimen was properly orientated and sent immediately to pathology for frozen section analysis, confirming negative surgical margins of at least 1 mm. Next the argon beam laser was used to extensively coagulate the cut surface of the renal cortex. The incision of the kidney (cortex and medullae) was then completely covered by either 5 ml of Floseal^®^ in 11 patients which was the agent of choice from December 2009 to February 2011 or 3 grams of Arista^®^ in 12 patients which was the agent of choice from March 2011 to the present. Into the renal defect, a large pledget of Surgicel^®^ was placed. Vertical mattress sutures, incorporating the Surgicel^®^ pledget, was placed to close the renal defect.

Complications were graded with Clavien-Dindo Classification and were noted to be either intra-operative or post-operative. Post-operative complications were classified as urological and nonurological (*e*.*g*., cardiac, gastrointestinal pulmonary, thromboembolic, incisional or other). Urological complication was defined as significant hemorrhage > 500 mL necessitating intervention or transfusion, urine leakage (drainage of greater than 50 mL daily for more than one week with fluid biochemistry compatible with urine) and acute renal failure (resulting in any dialysis, ureteral obstruction or kidney loss) [10]. In addition, serum creatinine levels were assessed four week after surgery and post-operative GFR was calculated.

Chi-square and T- student test was used to compare outcomes between the cohort of 11 patients who had Floseal^®^ and the 12 patients who had Arista^®^. *P* values less than 0.05 were considered to be statistically significant. All data were analyzed using SPSS software version 22.

## Results

Demographics, laboratory values and disease characteristics of the entire cohort are summarized in Table 1. The mean age of the entire cohort was 57.9 ± 10.4 years. The majority of patients were male (57%), Caucasian (52%) with left-sided lesions (78%). Median pre-operative size of renal mass measured on axial imaging was 4.3 cm (range 1.5 - 7 cm). Mean BMI was 30.2 ± 4.5 kg/m^2^ and mean pre-operative GFR was 82.0 ± 19.2 mL/min/1.73 m^2^. Regarding these demographics, laboratory values and disease characteristics, the Floseal^®^*vs*. Arista^®^ groups did not differ significantly.

**Table 1:** Demographics and preoperative data

**Variable**	**Total cohort (N = 23)**	**Floseal^®^ cohort (N = 11)**	**Arista^®^ cohort (N = 12)**	***p *value**
Mean age (±SD, years)	57.9 ± 10.4	55.0 ± 9.6	60.5 ± 10.9	0.45
Male:female	13:10	7:4	6:6	.51
Race				0.40
Caucasian	12 (52%)	5 (46%)	7 (58%)	
African American	3 (13%)	1 (8%)	2 (17%)	
Latinos	7 (31%)	5 (46%)	2 (17%)	
Others	1 (4%)	0 (0%)	1 (8%)	
Tobacco history	12 (52%)	6 (55%)	6 (50%)	0.82
Mean BMI (± SD, kg/m^2^)	30.2 ± 4.5	29.6 ± 2.8	30.8 ± 5.7	0.56
Mean Preoperative GFR (±SD, mL/min/1.73 m^2^)	82.0 ± 19.2	82.0 ± 25.3	82.0 ± 12.5	1.00
Side of renal mass (R/L)	5/18	1/10	4/8	0.15
Median size of renal mass (cm)	4.3 (range, 1.5-7 )	4.4 ± 1.8	4.2 ± 1.4	0.70

The median cool ischemia time was 30.8 minutes in the Floseal^®^ group compared to 35.4 minutes in the Arista^®^ group (*p* = 0.075). Mean intra-operative blood loss was 227 mL in the Floseal^®^ group compared to 250 mL in the Arista^®^ group (*p* = 0.68).

Post-operative disease characteristics and laboratory values of the entire cohort are summarized in Table 2. The majority of patients had clear cell variant of RCC (74%), pathologic stage T1a (70%) and Fuhrman grade 2 (55%). The previously described Bosniak cysts were found to be clear cell RCC. Disease characteristics were similar between the two groups. Mean post-operative GFR was 66.9 ± 21.8 mL/min/1.73 m^2^ in the Floseal cohort and 74.5 ± 15.4 mL/min/1.73 m^2^ in the Arista group, *p* = 0.34). Mean hospital stay between the Floseal^®^*vs*. Arista^®^ cohorts did not significantly differ (4.4 days *vs*. 4.5 days, *p* = 0.76, respectively).

**Table 2:** Oncologic and functional outcomes

**Variable**	**Total cohort (N = 23)**	**Floseal^®^ cohort (N = 11)**	**Arista^®^ cohort (N = 12)**	***p *value**
Histology				0.34
Clear cell	17 (74%)	9 (82%)	8 (66%)	
Papillary	2 (9%)	0 (%)	2 (17%)	
Chromophone	1 (4%)	1 (9%)	0 (0%)	
Oncocytoma	3 (13%)	1 (9%)	2 (17%)	
Pathologic stage				0.86
T1a	14 (70%)	7 (70%)	7 (70%)	
T1b	6 (30%)	3 (30%)	3 (30%)	
Furhman grade				0.14
1	8 (40%)	6 (60%)	2 (20%)	
2	11 (55%)	4 (40%)	7 (70%)	
3	1 (5%)	0 (0%)	1 (10%)	
4	0 (0%)	0 (0%)	0 (0%)	
Pos. surgical margin				N.A.
	0 (0%)	0 (0%)	0 (0%)	
Mean post-operative GFR (mL/min/1.73 m^2^)	70.9 ± 18.7	66.9 ± 21.8	74.5 ± 15.4	0.34
Hospital stay/days	4.5 ± 0.9	4.4 ± 1.2	4.5 ± 0.7	0.76

Seven complications (two intra-operative and five post-operative) were noted in 6 (26%) of the patients. The two intra-operative complications included transfusion for 500 mL blood loss (Clavien II) and small ipsilateral pneumothorax (Clavien I). The pnuemothorax was managed conservatively with complete resolution noted over two weeks. The five post-operative complications included prolonged urinary leak of 10 days (Clavien I) and readmission with 30 days for chest pain (Clavien I) (both in one patient), ileus = 1 (Clavien II), and >25% reduction in GFR-2 = 2 (Clavien I) (Table 3). No difference was noted in post-operative complications in patients in the Floseal^®^ group compared to the Arista^®^ group. No post-operative mortalities were noted. No recurrences have been documented with a mean follow-up of 18 months.

**Table 3:** Complications associated with NSS

**Complications**	**Total cohort (N = 23)**	**Floseal^®^ cohort (N = 11)**	**Arista cohort (N = 12)**	***p *value**
Urologic				
Excessive blood loss with transfusion	1	0	1	0.32
Prolonged urinary leak	1^~^	0	1	0.32
>25% reduction in GFR	2	2	0	0.12
Other^^^	0	0	0	N/A
Non-urologic				
Ileus	1	0	1	0.32
Wound infection	0	0	0	N/A
Other^*^	3^~^	1	2	0.59

^~,^ one patient had two complications (readmission within 30 days for chest pain with a negative evaluation and prolonged urinary drainage-drain removed on POD#10.

^^,^acute renal failure resulting in any dialysis, ureteral obstruction or kidney loss.

^*,^ cardiac, gastrointestinal pulmonary, thromboembolic, incisional or other.

N/A, not applicable.

## Discussion

Nephron-sparing surgery was initially used to treat patients with anatomically or functionally solitary kidney, bilateral renal masses or hereditary renal cell carcinoma or patients affected by comorbidities that might impair renal function, such as diabetes, hypertension, atherosclerosis, calculous renal disease, chronic pyelonephritis, ureteral reflux, renal artery stenosis and other causes of glomerulopathies [14,17]. Today, patients without any of the above conditions may be considered for NSS, since cancer specific survival and metastasis free survival are similar in all T1N0M0 renal tumors treated with NSS or radical nephrectomy [18-21]. Furthermore, NSS is associated with a decreased risk of chronic kidney disease, cardiovascular events and mortality when compared to radical nephrectomy [10].

Open NSS can be by far more complex than radical nephrectomy. Several decades ago, reports on open NSS described a greater risk of complications including: acute renal failure, urinary fistula, hemorrhage and others [14,22]. For example, Campbell *et al*. described complication rates after open NSS of 37% for symptomatic tumors and of 22% for incidental tumors [14]. Complication rates associated with NSS (*e*.*g*., overall morbidity rate, hospital stay, blood losses, and frequency of acute renal failure) have decreased significantly [23,24], which may be attributed to the greater experience of the urologist [25] or perhaps due to the incorporation of various new technologies (*i*.*e*., hemostatic agents, lasers, etc.) into the procedure. Interestingly, the use of NSS has increased only by 20% from 1995 to 2007 with 72% of Medicare beneficiaries with localized tumors being managed with radical nephrectomy [26]. With the comparable survival rates and decreasing morbidities, it is unclear why NSS remains underutilized in this population.

In our small cohort, we were able to compare the use of Floseal^®^ and Arista^®^ in this cohort. Increased risk of bleeding was not evident with the use of the new haemostatic agent, Arista^®^. Haemostatic agents such as Floseal^®^ or Arista^®^ in addition with an adequate surgical technique were adequate in maintaining post-operative haemostasis. No patient was noted to have a clinical post-operative bleed. Several benefits exist with Arista^®^. First, no mixing is required and thus the agent can be used immediately. Second, Arista^®^ is a synthetic haemostatic reagent and Floseal^®^ is made from human plasma, the latter may carry a risk for virus or prion infection. This is the first report of Arista^®^ being used in a NSS.

**Figure 1:**
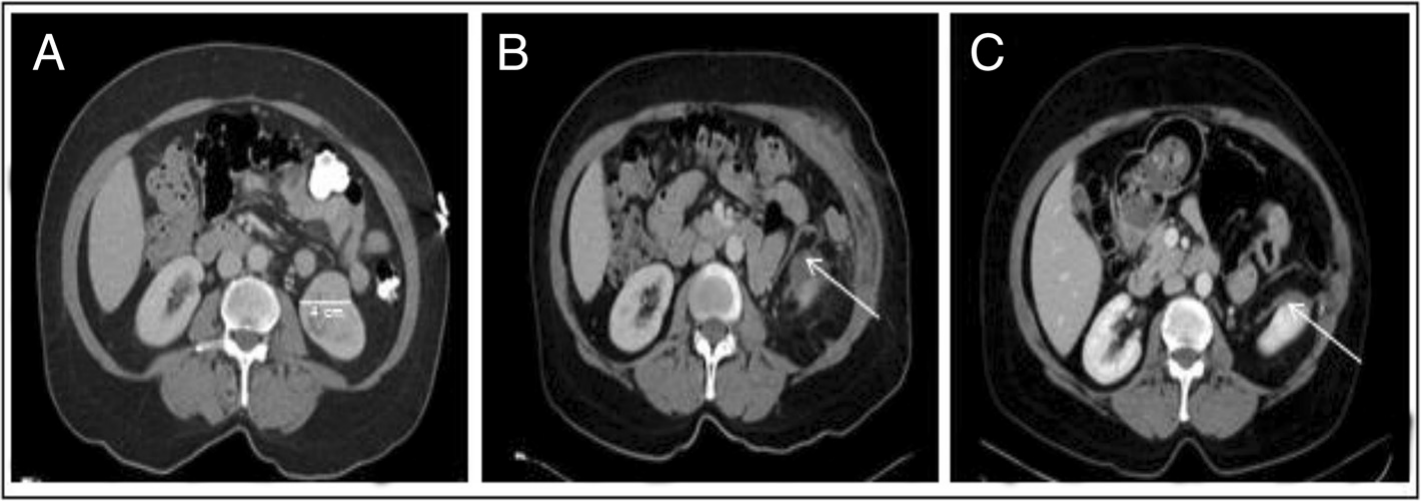
**Pre-operative and post-operative CT scan of patient who underwent NSS. A)** Pre-operative CT scan with intravenous contrast demonstrated 4 cm solid, exophytic mass in the lower pole of the left kidney. Patient underwent an open left NSS. **B)** Four weeks after NSS, patient presented to Emergency Department (ED) with abdominal pain. Patient was without fever/chills, elevated white blood cell count or an abnormal urinalysis. However, CT scan in the ED was interrupted as an abscess at the surgical site in the left kidney. Arrow illustrating fluid collection. **C)** Due to the unimpressive clinical scenario, no intervention was performed and a repeat CT scan of the abdomen 8 weeks after the visit to the ED noted healing surgical site. Arrow illustrating reduction in fluid collection and healing parenchyma.

Radiologic imaging after complex renal surgery may be problematic and thus urologist may have to clearly describe surgical technique to the radiologist interpreting post-operative scans. For example, we noticed on several occasions Surgicel^®^ pledgets have a space occupying low attenuation appearance, which could mimic an abscess (Figure 1). Thus we must stress that in the immediate post-operative setting (*i*.*e*., with 30 days), unless the patient has fever, elevated white blood cells count that caution must be exercised in over interpreting post-operative imaging.

Our study has several limitations. First, it is a small retrospective study from a single center performed by one surgeon. Next, our follow-up was limited (mean follow-up of 18 months) to reliably report any differences in oncologic outcomes. This short follow-up, however, is sufficient when assessing intra-operative and post-operative outcomes. Lastly, we devoted significant attention to the cut surface of the kidney (*i*.*e*., tying off lobar vessels, coagulating renal cortex with Argon laser in addition to placing a Surgicel^®^ pledget). Thus the true need for the haemostatic agent cannot be answered in this study.

## Conclusion

In conclusion, along with meticulous surgical technique, the use of either haemostatic agent (Floseal^®^ or Arista^®^) was not associated with high rate of intra-operative or post-operative haemorrhage. When requiring the application of a haemostatic agent during NSS, either haemostatic agent studied may be successfully employed.

## Abbreviations

CT: Computed tomography; MRI: Magnetic resonance imaging; GFR: Glomerular filtration rate; RCC: Renal cell carcinoma; NSS: Nephron sparing surgery; BMI: Body mass index.

## Competing interests

The authors declare that they have no competing interests.

## Authors’ contributions

DAP, MD - Study concept and design, drafting of manuscript. MMc, MD – Study concept and design, drafting of manuscript. MM, MD, PhD – Statistical analysis and drafting of manuscript. CJR, MD, MBA - Study concept and design, drafting of manuscript. All authors have read and approved the final manuscript.
